# Prevalence and correlates of destructive behaviors in the US Naval Surface Forces from 2010–2020

**DOI:** 10.1186/s40359-023-01134-1

**Published:** 2023-04-07

**Authors:** Kevin Lai, Jason T. Jameson, Dale W. Russell

**Affiliations:** 1 Leidos, Naval Health Research Center, 329 Ryne Road, San Diego, CA 92152 USA; 2 Commander, Naval Surface Force, U.S. Pacific Fleet, Coronado, CA USA; 3 Department of Psychiatry, Uniformed Services University of the Health Sciences, 4301 Jones Bridge Road, Bethesda, MD 20814 USA

**Keywords:** Suicide, Military, Navy, Domestic violence, Sexual assaults

## Abstract

**Purpose:**

To estimate the prevalence of domestic violence, sexual assault, and suicide for United States Navy (USN) personnel between 2010 and 2020 and identify potential associated factors.

**Methods:**

Official report data were used to calculate prevalence rates and odds ratios, accounting for sample and general USN population demographic data to assess differences in over- or underrepresentation of destructive behaviors.

**Results:**

Domestic violence and sexual assault offenders tended to be younger lower-ranked males. For sexual assaults, offenders were three times more likely to be senior to the victim, which was not the case for domestic violence. Females were overrepresented in terms of suicidal ideation and attempts relative to the USN population, while males accounted for more actual suicides. The relative rates of suicidal ideation and attempts for females exceeded those for males (i.e., comparing the sample rate against the USN male and female populations), but the sample proportion for completed suicides (compared to the USN population) were greater for males than for females. Those in the junior enlisted (E1–E3) paygrades exhibited greater odds of suicide attempts versus suicidal ideations relative to those in the Petty Officers (E4–E6) paygrades, although E4–E6s completed more suicides.

**Conclusion:**

The descriptive profile of destructive behaviors in a representative sample of USN personnel provides an overview of the possible factors associated with destructive behaviors and includes an exploration of the relational dynamics and nature of the incidents. The results suggest that sexual assault and domestic violence are characterized by unique relational dynamics and that these destructive behaviors should not necessarily be classified together as male-oriented aggressions (i.e., mainly perpetrated by males against female victims). Those in the E1–E3 and E4–E6 paygrades displayed different patterns in suicidal ideation, attempts, and actual suicides. The results highlight individual characteristics to help inform the development of targeted policies, practices, and interventions for military and other hierarchical organizations (e.g., police).

## Introduction

Destructive behaviors have been broadly defined as conduct that results in or presents imminent danger to the person exhibiting the behavior to others (e.g., co-workers, friends, and family members) or to property [1, 2]. In the commercial sector, destructive behaviors can have negative externalities such as reduced productivity, declines in customer service, and lost profits [3]. In a military context, however, destructive behaviors also have national security implications as they undermine unit cohesion, combat readiness, and ultimately warfighting capabilities [4].

Military personnel face a number of environmental (e.g., austere work settings) and occupational (e.g., combat) stressors that can result in a multitude of negative mental health outcomes, which have increased in frequency over the last two decades [5]. Over the same period of time, the US military has experienced an increase in the incidence rates of destructive behaviors, especially suicide-related behaviors [6], which suggests a relationship between mental health and destructive behaviors [7]. The impact of destructive behaviors extends beyond the individual to the group, potentially undermining military team cohesion [8]. For instance, the prevalence of destructive behaviors has increased in military units that experienced a suicide [9, 10].

Despite a growing interest in destructive behaviors in military populations, there is a dearth of research in this domain, especially in relation to naval personnel. In response, the US Navy (USN) instituted an initiative called the Culture of Excellence, which aims to address destructive behaviors, among other things, “by fostering psychological, physical and emotional toughness; promoting organizational trust and transparency; and ensuring inclusion and connectedness among every sailor, family member and civilian throughout their Navy journey” [11]. A key facet of fostering Culture of Excellence is understanding the general scope and contributing factors driving destructive behaviors. To that end, this research examines destructive behavior data from USN personnel between 2010 and 2020. The goal is to establish prevalence rates and explore possible factors associated with destructive behaviors. These findings will help assess the long-term effects of sustained military operations on an all-volunteer force and help inform the development of prevention/intervention efforts to enhance the health and well-being of servicemembers.

## Reported destructive behaviors

### Domestic violence

The US Department of Defense (DoD) defines domestic violence as “an offense that involves the use, attempted use, or threatened use of force or violence against a person, or a violation of a lawful order issued for the protection of a: (1) person who is a current or former spouse, (2) person with whom the abuser shares a child in common, or (3) current or former intimate partner with whom the abuser shares or has shared a common domicile” [12]. For civilians, the prevalence rates for those who have experienced some form of domestic violence in their lifetime can reach upwards of 25% for females and 14% for males [13], compared to up to 33% for females and 17% for males in the military [14].

Some risk factors, such as previous violence perpetration and substance abuse, are common to both the general and military populations [15], but there is evidence that military-specific experiences contribute to the higher rates of domestic violence [4]. For instance, combat deployment experiences (e.g., having killed/wounded others) may increase a servicemember’s likelihood to exhibit domestic violence behaviors [14].

The military has a hypermasculine mystique and previous research has focused on male as perpetrators and female as victims, which may present a limited view on otherwise complex relational dynamics [14, 16]. Data availability has also limited previous efforts to understand the prevalence and causes of domestic violence in military populations [17, 18].

### Sexual assault

The DoD defines sexual assault as “intentional sexual contact characterized by use of force, threats, intimidation, or abuse of authority or when the victim does not or cannot consent” [19]. In the general population, estimates indicate that 28–33% of females and 12–18% of males experience sexual abuse during their lifetime [20]. Within the US military, depending on the sample, estimates range from 15–49% for females to 2–23% for males [21, 22]. A study with a more recent sample estimated that 6% of female and 0.7% of male US servicemembers have experienced a sexual assault [23]. Although sexual assault is an issue for all servicemembers, there is a greater percentage of cases involving female victims [24]. Some have theorized that this can be due to several factors, including lower sociocultural and organizational power possessed by females, which can be amplified in a military setting given its often hypermasculine leaning [16, 21].

In the context of sexual assault, substance use (i.e., namely alcohol) is often a contributing factor and associated with both offender and victim consumption [25–28]; for example, one DoD report noted that alcohol was involved in over 50% of sexual assault cases at military academies [29]. Alcohol use is also especially prevalent in military populations due to various factors, including peer pressure, a drinking culture, easy access to alcohol, and operational/environmental stressors that compel use as coping mechanism [30, 31].

Sexual assault among servicemembers and veterans, especially females, can lead to numerous negative outcomes, such as post-traumatic stress disorder [32, 33], poor servicemember retention [34], degraded unit cohesion [35], and degraded combat readiness [36]. Although the DoD has enacted far-reaching policies and practices to reduce sexual assaults, the issues persist for reasons still not fully understood [23, 37]. As such, military-salient research is needed to: (1) identify where problems exist and who is affected by them; (2) characterize the magnitude of those problems; (3) identify factors associated with those problems; and (4) identify military-relevant prevention/intervention strategies [38]. This research seeks to contribute to these topics within the context of a relatively understudied, relative to US Army and Marine Corps frontline personnel, yet at-risk military population: the USN’s Surface Force.

### Suicide behaviors

Suicide is a global concern, with approximately one million people in the world taking their own lives per year [39]. In the US, suicide is the tenth leading cause of death in the general population, but the second leading cause of death for those aged 10–34 [40], and the second leading cause in the military [41]. To provide more context, the global suicide rate is 13.3 per 100,000 compared to 17.4 for the general US population, but 21.9 for the US military’s Active Component, 25.7 for the Reserve Component (i.e., Federally-controlled reserves), and 29.1 for the National Guard (i.e., State-controlled reserves) per 100,000 [42]. Furthermore, since 2001, military suicides have occurred at a rate four times greater than combat-related deaths [6].

Suicide-related behaviors are the product of a complex system of interacting causes some of which include demographic characteristics [43, 44]. In 2019, males in the US were three times more likely to die by suicide than females, although females were more likely to exhibit suicidal ideations and attempts. There are a number of possible explanations for such differences. For instance, females may benefit from greater levels of social support compared to males [45]. Males may also be more comfortable with, and have greater access to, weapons [46]. Within the military, studies focused on US Army soldiers found primary demographic risk factors to include being a white male aged 17–19 [47]. Race may also be a contributing factor due to associated cultural and socioeconomic factors that impact resource availability (e.g., access to care) and social support (e.g., via religious affiliations) [44, 46, 47]. For instance, African Americans may more readily engage personal support systems (e.g., attend religious activities) which may act as a protective factor against suicide-related behaviors [47].

Taken together, the large body of suicide research signals a complex interplay among risk and protective factors associated with suicidal behaviors. As with domestic violence and sexual assaults, the DoD has undertaken a number of efforts to stem suicide-related behaviors, yet the problem persists. As such, problems persist at different rates across military groups (e.g., infantrymen vs. medical personnel and Navy vs. Army), it is important to surveil military sub-groups as to better monitor and understand the factors relevant to each group in order to best shape policies, allocate resources and develop support programs [48]. To that end, this paper seeks to provide a deeper understanding of destructive behavior outcomes in naval context by leveraging a unique longitudinal dataset.

## Methods

### Data source

The data for this study were obtained from USN Operational Reports (OPREP-3). These reports are submitted by subordinate units to provide timely awareness to higher-level commands when special destructive behavior related events occur (e.g., a suicide, domestic violence, harassment, an assault, and suicide-related behavior) [49]. These reports contain no personally identifiable information and capture only the basic facts about an incident, which include: incident date/time, reporting command name, brief text synopsis of the incident, offender’s details (e.g., gender, age, paygrade, and race/ethnicity), victim’s details (e.g., gender, age, paygrade, and race/ethnicity), incident type (e.g., legal/illicit substance-related), description of any weapons involved, whether a law enforcement arrest was made, and geographic location. All the available OPREP-3 data from 2010 to 2020 were included in the study. There were no inclusion or exclusion criteria.

### Statistical analysis

To assess the sample’s representativeness, the demographics (paygrade, gender, age, and race) reported in the OPREP-3 data were compared to the USN’s annual populations between 2010 and 2020 [50]. Chi-square tests were used to determine the magnitude and statistical significance of differences across key demographic characteristics from the OPREP-3 data as compared to the entire USN’s population demographics for each year.

Racial categories comprised white, black, and other as the cases of non-white, non-black were smaller [48]. Paygrades were categorized as E1–E3 (Junior Enlisted), E4–E6 (Petty Officers), E7–E9 (Chiefs), and Officers. Following the precedent of USN population reporting [50], age groups were categorized as: < 25, 26–30, 31–35, 36–40, and > 41. Values that did not correspond to the above categories were categorized as Other, which also includes missing data. Chi-square tests comparing the OPREP-3 data to the available demographics data only included data in the defined categories (not including Other).

Logistic regression models were used to calculate odds ratios (OR). Specifically, for the suicide data, the categorizations of suicide attempts and ideation allowed for the relationship assessment between servicemembers’ suicide attempts as compared to ideation across the four main demographic categories (i.e., gender, race, age, and paygrade). ORs and 95% confidence intervals were calculated for each of the above dimensions. Data were prepared and analyzed with R 4.1.1 and the dplyr, ggplot2, ggparallel packages [51–54]

## Results

### Domestic violence

Table 1 provides an overview of the domestic violence incident data by year. The results show that in general, across the years, the number of female and male offenders reflect the observed proportions in the overall USN. With respect to age, there were more offenders aged 25 and under (48.41%) relative to the Navy’s general population of those aged 25 and under (42.07%), *X*^2^(4, *N* = 4674) = 258.01, *p* < 0.001. Regarding race, there was a consistently higher proportion of black (46.80%) than white (53.20%) offenders when taking into account the overall demographics, which was reliably different from the expected proportion based on the overall demographic breakdown (21.94% white versus 78.06% black), *X*^2^(1, *N* = 3960) = 1440.17, *p* < 0.001. Regarding paygrades, more cases were reported for those in the paygrades of E4–E6 than in any other paygrade group, *X*^2^(3, *N* = 3896) = 608.15, *p* < 0.001. For victims of domestic violence, there was generally a higher rate of being black, female, under age 25, and in the E4–E6 paygrades.

**Table 1:** Domestic violence: distribution of selected characteristics for the total sample and by demographics across the years

	2010		2011		2012		2013		2014		2015		2016	
	N	%	N	%	N	%	N	%	N	%	N	%	N	%
Offender														
Gender	*p* *> 0.05*		*p* *> 0.05*		*p* *<* *0.05*		*p* *<* *0.01*		*p* *> 0.05*		*p* *> 0.05*		*p* *<* *0.001*	
Female	70	16.91	117	19.31	104	20.16	97	22.66	84	19.27	100	19.65	116	25.49
Male	344	83.09	489	80.69	412	79.84	331	77.34	352	80.73	409	80.35	339	74.51
Other	0		1		3		2		4		1		6	
Age	*p* *<* *0.001*		*p* *<* *0.001*		*p* *<* *0.001*		*p* *<* *0.001*		*p* *<* *0.001*		*p* *<* *0.001*		*p* *<* *0.05*	
25 and under	213	54.34	306	56.46	223	46.95	186	45.81	195	48.51	236	50.00	181	41.90
26–30	100	25.51	121	22.32	145	30.53	109	26.85	103	25.62	128	27.12	124	28.70
31–35	42	10.71	68	12.55	62	13.05	60	14.78	62	15.42	55	11.65	58	13.43
36–40	26	6.63	26	4.80	36	7.58	39	9.61	29	7.21	33	6.99	44	10.19
41+	11	2.81	21	3.87	9	1.89	12	2.96	13	3.23	20	4.24	25	5.79
Other	22		65		44		24		38		38		29	
Race	*p* *<* *0.001*		*p* *<* *0.001*		*p* *<* *0.001*		*p* *<* *0.001*		*p* *<* *0.001*		*p* *<* *0.001*		*p* *<* *0.001*	
White	175	54.18	240	52.75	240	54.18	161	48.79	197	57.77	209	50.24	204	53.83
Black	148	45.82	215	47.25	203	45.82	169	51.21	144	42.23	207	49.76	175	46.17
Other	91		152		122		100		99		94		82	
Rank	*p* *<* *0.001*		*p* *<* *0.001*		*p* *<* *0.001*		*p* *<* *0.001*		*p* *<* *0.001*		*p* *<* *0.001*		*p* *<* *0.001*	
E1–E3	82	25.23	153	33.19	84	22.52	38	11.91	63	19.03	81	21.26	71	20.29
E4–E6	225	69.23	277	60.09	257	68.90	243	76.18	238	71.90	249	65.35	228	65.14
E7–E9	7	2.15	23	4.99	19	5.09	28	8.78	24	7.25	33	8.66	37	10.57
Officer/CWO	11	3.38	8	1.74	13	3.49	10	3.13	6	1.81	18	4.72	14	4.00
Other	89		146		146		111		109		129		111	
Victim														
Gender	*p* *<* *0.001*		*p* *<* *0.001*		*p* *<* *0.001*		*p* *<* *0.001*		*p* *<* *0.001*		*p* *<* *0.001*		*p* *<* *0.001*	
Female	344	83.29	481	80.03	391	78.83	332	78.12	347	80.70	403	80.92	321	74.31
Male	69	16.71	120	19.97	105	21.17	93	21.88	83	19.30	95	19.08	111	25.69
Other	1		6		23		5		10		12		29	
Age	*p* *<* *0.001*		*p* *<* *0.001*		*p* *<* *0.001*		*p* *<* *0.001*		*p* *<* *0.001*		*p* *<* *0.001*		*p* *<* *0.001*	
25 and under	186	60.59	270	60.27	206	51.89	181	52.31	190	51.91	232	55.50	186	50.00
26–30	69	22.48	101	22.54	108	27.20	79	22.83	87	23.77	112	26.79	102	27.42
31–35	28	9.12	53	11.83	48	12.09	49	14.16	49	13.39	40	9.57	45	12.10
36–40	14	4.56	16	3.57	30	7.56	24	6.94	25	6.83	25	5.98	23	6.18
41+	10	3.26	8	1.79	5	1.26	13	3.76	15	4.10	9	2.15	16	4.30
Other	107		159		122		84		74		92		89	
Race	*p* *<* *0.001*		*p* *<* *0.001*		*p* *<* *0.001*		*p* *<* *0.001*		*p* *<* *0.001*		*p* *<* *0.001*		*p* *<* *0.001*	
White	143	63.00	197	54.87	189	55.26	156	53.98	173	57.67	189	53.24	192	57.14
Black	84	37.00	162	45.13	153	44.74	133	46.02	127	42.33	166	46.76	144	42.86
Other	187		248		177		141		140		155		125	
Rank	*p* *<* *0.001*		*p* *<* *0.001*		*p* *<* *0.001*		*p* *<* *0.001*		*p* *<* *0.001*		*p* *<* *0.001*		*p* *<* *0.001*	
E1–E3	50	36.76	81	35.37	45	8.67	38	23.46	31	20.39	58	30.53	45	26.16
E4–E6	79	58.09	142	62.01	134	25.82	118	72.84	109	71.71	122	64.21	114	66.28
E7–E9	6	4.41	2	0.87	9	1.73	4	2.47	9	5.92	8	4.21	6	3.49
Officer/CWO	1	0.74	4	1.75	0	0.00	2	1.23	3	1.97	2	1.05	7	4.07
Other	278		378		331		268		288		320		289	

	2017		2018		2019		2020		Incidents total		Navy average demographics 2010–2020
	N	%	N	%	N	%	N	%	N	%	%
Offender											
Gender	*p* *> 0.05*		*p* *> 0.05*		*p* *> 0.05*		*p* *> 0.05*		*p* *<* *0.001*		
Female	89	20.99	90	21.13	79	18.72	82	21.58	1028	20.49	18.26
Male	335	79.01	336	78.87	343	81.28	298	78.42	3988	79.51	81.74
Other	4		16		10		11		58		
Age	*p* *<* *0.05*		*p* *<* *0.01*		*p* *<* *0.001*		*p* *<* *0.001*		*p* *<* *0.001*		
25 and under	166	42.13	179	43.77	197	52.25	168	48.41	2250	48.41	42.07
26–30	108	27.41	107	26.16	71	18.83	95	27.38	1211	26.05	23.06
31–35	60	15.23	67	16.38	70	18.57	50	14.41	654	14.07	15.21
36–40	43	10.91	42	10.27	26	6.90	28	8.07	372	8.00	10.86
41+	17	4.31	14	3.42	13	3.45	6	1.73	161	3.46	8.81
Other	34		33		55		44		426		
Race	*p* *<* *0.001*		*p* *<* *0.001*		*p* *<* *0.001*		*p* *<* *0.001*		*p* *<* *0.001*		
White	201	57.43	212	58.56	147	48.36	155	55.16	2095	53.20	78.06
Black	149	42.57	150	41.44	157	51.64	126	44.84	1843	46.80	21.94
Other	78		80		128		110		1136		
Rank	*p* *<* *0.001*		*p* *<* *0.001*		*p* *<* *0.001*		*p* *<* *0.001*		*p* *<* *0.001*		
E1–E3	67	19.88	56	16.05	90	26.47	81	26.21	866	22.35	22.57
E4–E6	217	64.39	257	73.64	210	61.76	195	63.11	2596	66.99	51.40
E7–E9	37	10.98	21	6.02	27	7.94	18	5.83	274	7.07	9.30
Officer/CWO	16	4.75	15	4.30	13	3.82	15	4.85	139	3.59	16.73
Other	91		93		92		82		1199		
Victim											
Gender	*p* *<* *0.001*		*p* *<* *0.001*		*p* *<* *0.001*		*p* *<* *0.001*		*p* *<* *0.001*		
Female	317	81.49	310	76.54	307	79.53	295	82.17	3848	79.60	18.26
Male	72	18.51	95	23.46	79	20.47	64	17.83	986	20.40	81.74
Other	39		37		46		32		240		
Age	*p* *<* *0.001*		*p* *<* *0.001*		*p* *<* *0.001*		*p* *<* *0.001*		*p* *<* *0.001*		
25 and under	163	48.95	184	51.69	171	57.97	137	51.89	2106	53.97	42.07
26–30	92	27.63	91	25.56	60	20.34	76	28.79	977	25.04	23.06
31–35	41	12.31	40	11.24	37	12.54	29	10.98	459	11.76	15.21
36–40	29	8.71	30	8.43	21	7.12	17	6.44	254	6.51	10.86
41+	8	2.40	11	3.09	6	2.03	5	1.89	106	2.72	8.81
Other	95		86		137		127		1172		
Race	*p* *<* *0.001*		*p* *<* *0.001*		*p* *<* *0.001*		*p* *<* *0.001*		*p* *<* *0.001*		
White	186	62.63	189	60.97	115	50.44	116	58.00	1845	56.89	78.06
Black	111	37.37	121	39.03	113	49.56	84	42.00	1398	43.11	21.94
Other	131		132		204		191		1831		
Rank	*p* *<* *0.001*		*p* *<* *0.001*		*p* *<* *0.001*		*p* *<* *0.001*		*p* *<* *0.001*		
E1–E3	41	26.11	38	22.89	55	33.74	25	17.48	507	27.29	22.57
E4–E6	102	64.97	116	69.88	102	62.58	104	72.73	1242	66.85	51.40
E7–E9	10	6.37	9	5.42	4	2.45	4	2.80	71	3.82	9.30
Officer/CWO	4	2.55	3	1.81	2	1.23	10	6.99	38	2.05	16.73
Other	271		276		269		248		3216		

Chi square test *p* value indicates whether the null hypothesis that there is no significant difference from the Navy population can be rejected

### Sexual assaults

Table 2 provides an overview of sexual assault incidents by year. There were significantly more male offenders than females (96.72% of the offenders were male), *X*^2^(1, *N* = 5310) = 800.39, *p* < 0.001. For age, those aged 25 and under constituted the majority of offenders (58.87%), which was higher in proportion to USN demographics, *X*^2^(4, *N* = 5310) = 518.46, *p* < 0.001. With respect to race, white sailors constituted the majority of offenders (64.56%), but the proportion was lower than what would be expected for the overall population, *X*^2^(1, *N* = 3533) = 375.96, *p* < 0.001. Regarding paygrades, most offenders were E1–E3 (33.27%) and E4–E6 (56.18%), *X*^2^(3, *N* = 3156) = 326.14, *p* < 0.001.

**Table 2:** Sexual assault: distribution of selected characteristics for the total sample and by demographics across the years

	2010		2011		2012		2013		2014		2015		2016	
	N	%	N	%	N	%	N	%	N	%	N	%	N	%
Offender														
Gender	*p* *<* *0.001*		*p* *<* *0.001*		*p* *<* *0.001*		*p* *<* *0.001*		*p* *<* *0.001*		*p* *<* *0.001*		*p* *<* *0.001*	
Female	3	1.24	5	1.54	8	2.03	16	3.64	7	1.38	10	2.00	18	3.22
Male	239	98.76	319	98.46	387	97.97	424	96.36	501	98.62	489	98.00	541	96.78
Other	12		9		4		33		14		26		24	
Age	*p* *> 0.05*		*p* *<* *0.01*		*p* *<* *0.001*		*p* *<* *0.001*		*p* *<* *0.001*		*p* *<* *0.001*		*p* *<* *0.001*	
25 and under	93	52.54	110	53.40	157	55.09	196	60.68	188	51.65	203	55.46	255	59.58
26–30	36	20.34	38	18.45	54	18.95	56	17.34	91	25.00	70	19.13	90	21.03
31–35	20	11.30	34	16.50	33	11.58	48	14.86	46	12.64	48	13.11	43	10.05
36–40	17	9.60	16	7.77	24	8.42	14	4.33	24	6.59	28	7.65	30	7.01
41+	11	6.21	8	3.88	17	5.96	9	2.79	15	4.12	17	4.64	10	2.34
Other	77		127		114		150		158		159		155	
Race	*p* *<* *0.001*		*p* *<* *0.001*		*p* *<* *0.001*		*p* *<* *0.001*		*p* *<* *0.001*		*p* *<* *0.001*		*p* *<* *0.001*	
White	99	65.13	113	57.95	156	61.18	173	60.70	200	62.89	234	67.44	267	62.09
Black	53	34.87	82	42.05	99	38.82	112	39.30	118	37.11	113	32.56	163	37.91
Other	102		138		144		188		204		178		153	
Rank	*p* *<* *0.001*		*p* *<* *0.001*		*p* *<* *0.01*		*p* *<* *0.001*		*p* *<* *0.001*		*p* *<* *0.001*		*p* *<* *0.001*	
E1–E3	58	34.94	68	34.00	51	26.29	30	28.85	52	27.96	66	29.73	124	35.94
E4–E6	85	51.20	104	52.00	114	58.76	65	62.50	107	57.53	122	54.95	188	54.49
E7–E9	14	8.43	18	9.00	14	7.22	6	5.77	21	11.29	23	10.36	19	5.51
Officer/CWO	9	5.42	10	5.00	15	7.73	3	2.88	6	3.23	11	4.95	14	4.06
Other	88		133		205		369		336		303		238	
Victim														
Gender	*p* *<* *0.001*		*p* *<* *0.001*		*p* *<* *0.001*		*p* *<* *0.001*		*p* *<* *0.001*		*p* *<* *0.001*		*p* *<* *0.001*	
Female	61	85.92	110	86.61	122	91.04	107	83.59	202	85.96	217	87.85	274	87.54
Male	10	14.08	17	13.39	12	8.96	21	16.41	33	14.04	30	12.15	39	12.46
Other	183		206		266		345		287		275		270	
Age	*p* *<* *0.001*		*p* *<* *0.001*		*p* *<* *0.001*		*p* *<* *0.001*		*p* *<* *0.001*		*p* *<* *0.001*		*p* *<* *0.001*	
25 and under	182	81.61	218	79.27	277	79.37	333	81.82	406	83.71	384	80.17	446	83.52
26–30	28	12.56	43	15.64	49	14.04	46	11.30	50	10.31	53	11.06	60	11.24
31–35	7	3.14	10	3.64	11	3.15	19	4.67	17	3.51	27	5.64	16	3.00
36–40	3	1.35	3	1.09	9	2.58	6	1.47	11	2.27	12	2.51	9	1.69
41+	3	1.35	1	0.36	3	0.86	3	0.74	1	0.21	3	0.63	3	0.56
Other	31		58		50		66		37		46		49	
Race	*p* *> 0.05*		*p* *> 0.05*		*p* *> 0.05*		*p* *> 0.05*		*p* *> 0.05*		*p* *> 0.05*		*p* *<* *0.01*	
White	146	79.78	167	76.96	219	75.52	253	76.67	306	76.12	296	74.19	323	72.10
Black	37	20.22	50	23.04	71	24.48	77	23.33	96	23.88	103	25.81	125	27.90
Other	71		116		109		143		120		126		135	
Rank	*p* *<* *0.001*		*p* *<* *0.001*		*p* *<* *0.001*		*p* *<* *0.001*		*p* *<* *0.001*		*p* *<* *0.001*		*p* *<* *0.001*	
E1–E3	123	59.71	142	51.08	182	54.01	193	52.16	206	46.92	215	50.59	268	52.45
E4–E6	78	37.86	124	44.60	144	42.73	166	44.86	222	50.57	190	44.71	222	43.44
E7–E9	0	0.00	1	0.36	2	0.59	4	1.08	2	0.46	8	1.88	1	0.20
Officer/CWO	5	2.43	11	3.96	9	2.67	7	1.89	9	2.05	12	2.82	20	3.91
Other	48		55		62		103		83		100		72	

	2017		2018		2019		2020		Incidents total		Navy average demographics 2010–2020
	N	%	N	%	N	%	N	%	N	%	%
Offender											
Gender	*p* *<* *0.001*		*p* *<* *0.001*		*p* *<* *0.001*		*p* *<* *0.001*		*p* *<* *0.001*		
Female	37	6.48	21	3.49	33	5.28	16	2.93	174	3.28	18.26
Male	534	93.52	580	96.51	592	94.72	530	97.07	5136	96.72	81.74
Other	34		45		112		120		433		
Age	*p* *<* *0.001*		*p* *<* *0.001*		*p* *<* *0.001*		*p* *<* *0.001*		*p* *<* *0.001*		
25 and under	283	61.52	303	61.34	270	66.50	235	60.88	2293	58.87	42.07
26–30	101	21.96	102	20.65	72	17.73	73	18.91	783	20.10	23.06
31–35	49	10.65	47	9.51	33	8.13	42	10.88	443	11.37	15.21
36–40	17	3.70	25	5.06	19	4.68	27	6.99	241	6.19	10.86
41+	10	2.17	17	3.44	12	2.96	9	2.33	135	3.47	8.81
Other	145		152		331		280		1848		
Race	*p* *<* *0.001*		*p* *<* *0.001*		*p* *<* *0.001*		*p* *<* *0.001*		*p* *<* *0.001*		
White	307	70.41	332	71.24	226	61.08	174	62.37	2281	64.56	78.06
Black	129	29.59	134	28.76	144	38.92	105	37.63	1252	35.44	21.94
Other	169		180		367		387		2210		
Rank	*p* *<* *0.001*		*p* *<* *0.001*		*p* *<* *0.001*		*p* *<* *0.001*		*p* *<* *0.001*		
E1–E3	130	33.77	118	29.28	196	38.66	157	35.36	1050	33.27	22.57
E4–E6	222	57.66	249	61.79	269	53.06	248	55.86	1773	56.18	51.40
E7–E9	19	4.94	19	4.71	29	5.72	19	4.28	201	6.37	9.30
Officer/CWO	14	3.64	17	4.22	13	2.56	20	4.50	132	4.18	16.73
Other	220		243		230		222		2587		
Victim											
Gender	*p* *<* *0.001*		*p* *<* *0.001*		*p* *<* *0.001*		*p* *<* *0.001*		*p* *<* *0.001*		
Female	249	81.64	235	84.53	289	83.05	227	82.85	2093	85.12	18.26
Male	56	18.36	43	15.47	59	16.95	47	17.15	366	14.88	81.74
Other	300		368		389		392		3284		
Age	*p* *<* *0.001*		*p* *<* *0.001*		*p* *<* *0.001*		*p* *<* *0.001*		*p* *<* *0.001*		
25 and under	458	81.64	465	79.49	514	83.85	430	79.93	4113	81.46	42.07
26–30	75	13.37	82	14.02	71	11.58	71	13.20	628	12.44	23.06
31–35	18	3.21	23	3.93	21	3.43	19	3.53	188	3.72	15.21
36–40	9	1.60	8	1.37	4	0.65	13	2.42	87	1.72	10.86
41+	1	0.18	7	1.20	3	0.49	5	0.93	33	0.65	8.81
Other	44		61		124		128		694		
Race	*p* *<* *0.01*		*p* *> 0.05*		*p* *<* *0.05*		*p* *<* *0.001*		*p* *<* *0.001*		
White	339	72.90	392	78.71	348	73.89	253	69.13	3042	74.76	78.06
Black	126	27.10	106	21.29	123	26.11	113	30.87	1027	25.24	21.94
Other	140		148		266		300		1674		
Rank	*p* *<* *0.001*		*p* *<* *0.001*		*p* *<* *0.001*		*p* *<* *0.001*		*p* *<* *0.001*		
E1–E3	222	44.76	262	48.88	311	51.07	283	52.70	2407	50.74	22.57
E4–E6	248	50.00	253	47.20	284	46.63	234	43.58	2165	45.64	51.40
E7–E9	5	1.01	4	0.75	2	0.33	7	1.30	36	0.76	9.30
Officer/CWO	21	4.23	17	3.17	12	1.97	13	2.42	136	2.87	16.73
Other	109		110		128		129		999		

Chi square test p value indicates whether the null hypothesis that there is no significant difference from the Navy population can be rejected

Females represented the majority (85.12%) of the sexual assault victims, significantly higher in proportion to the Navy demographics (18.26%), *X*^2^(1, *N* = 5648) = 16,314.03, *p* < 0.001. The majority (81.46%) of victims were aged 25 and under, which is also disproportionately high relative to the USN’s overall population, *X*^2^(4, *N* = 5049) = 3309.34, *p* < 0.001. Between 2010 and 2020, the white and black proportion for victims reflected the Navy demographics except in recent years (after 2016). Sailors in the E1–E3 paygrades constituted the majority (50.74%) of sexual assault victims, which was much higher in proportion to the overall demographics, *X*^2^(3, *N* = 4744) = 2618.12, *p* < 0.001.

### Relational dynamics

**Fig. 1:**
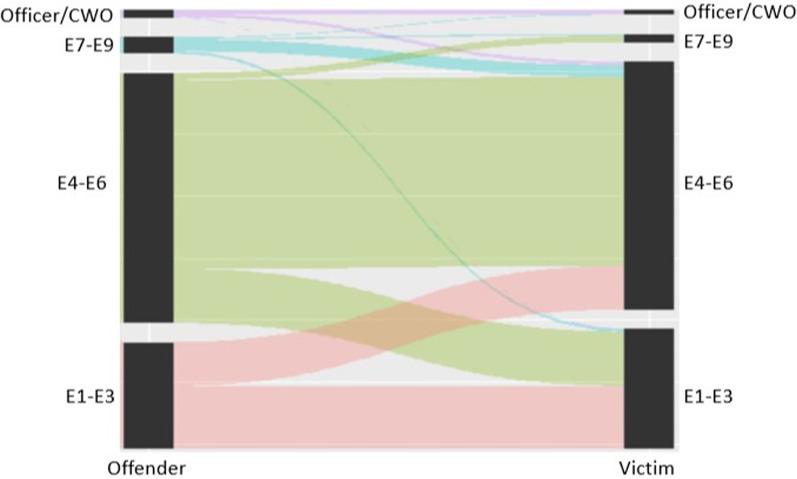
Domestics violence parallel plot

**Fig. 2:**
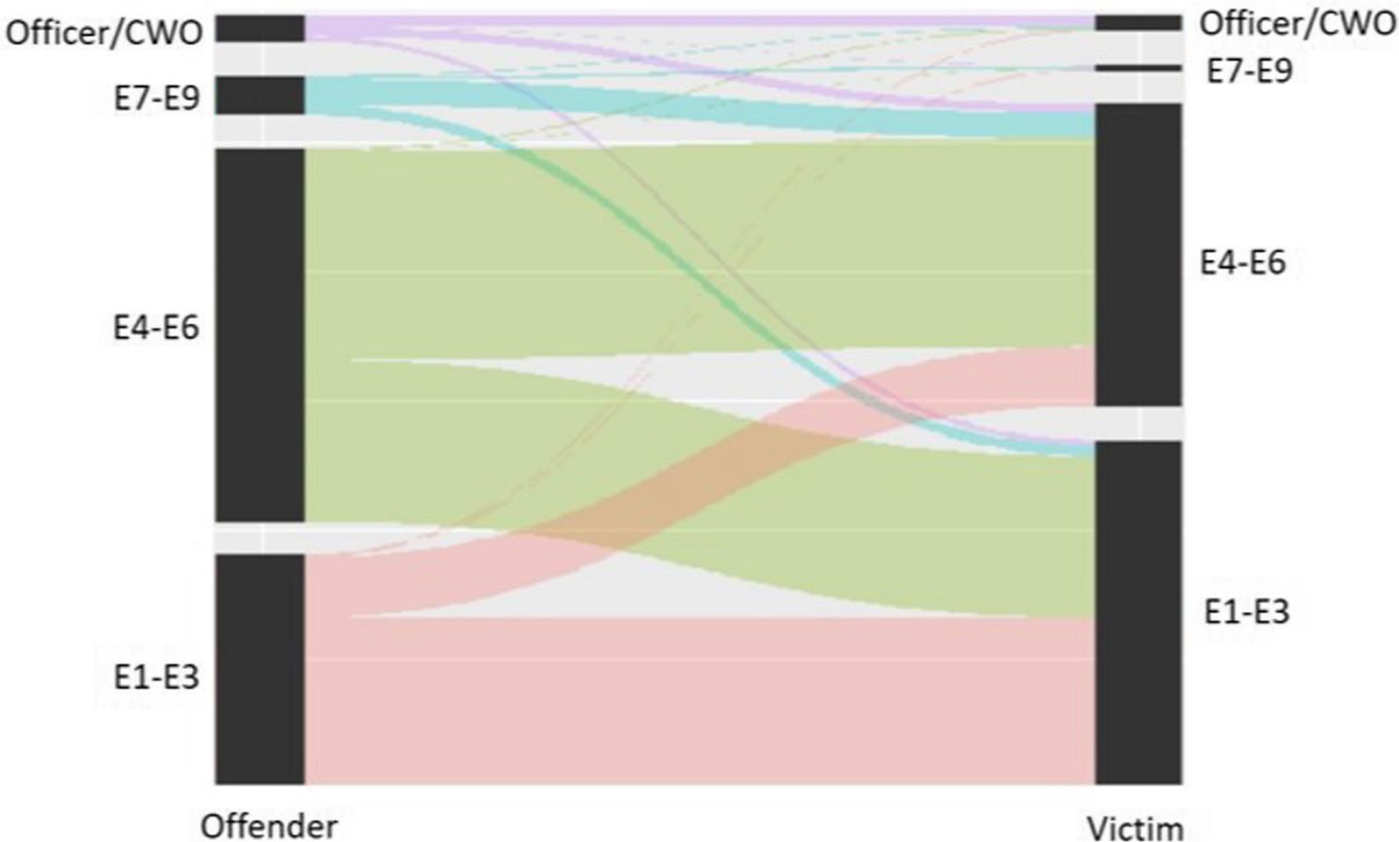
Sexual assault parallel plot

Figures 1 and 2 are parallel charts of the non-missing domestic violence and sexual assault incident data in relation to paygrade level. The relationship between the offender and victim was categorized as *senior* if the offender’s paygrade category was higher than the victim’s (e.g., E4–E6 vs. E1–E3), and as *junior* if the paygrade category was lower. For domestic violence the majority of offenders and victims were from the same paygrade category (67.36%). Of the remaining cases, 19.06% of cases were senior and 13.58% were junior (*X*^2^(1, *N* = 250) = 7.06, *p* < 0.01). For offender and victim relations in sexual assault cases, most of the incidents (58.31%) involved sailors in the same paygrade category. However, offenders were three times more likely to be senior to the victim than junior (31.74% versus 9.95% respectively, *X*^2^(1, *N* = 1081) = 295.31, *p* < 0.001).

### Alcohol involvement

Alcohol was associated with 38% of the domestic violence incidents. Of these, more incidents were reported of an offender with alcohol (33.81%) than of the victim with alcohol (25.18%) (*X*^2^(1, *N* = 7377) = 65.19, *p* < 0.001). Overall, in 21.58% reported incidents, there was alcohol use in both parties. Regarding age, 4% of the offenders using alcohol were 21 or younger while 16% of the victims who used alcohol were 21 or younger.

Regarding alcohol involvement in sexual assault, there were more reports of perpetrators consuming alcohol than of victims consuming alcohol (54.99% vs. 51.48%, *X*^2^(1, *N* = 8848) = 10.81, *p* = 0.001); the probability that one or the other had used alcohol was 59.92%. Regarding age, 12% of the sexual assault offenders using alcohol were 21 or younger while 27% of the victims who used alcohol were aged 21 or under.

### Suicide behaviors

#### Suicidal ideation

Table 3 provides an overview of the suicide-related incident data by year; of note, the Atlantic Fleet only began capturing suicidal ideation related data comparable to that of the Pacific Fleet’s 2010–2020 data. There was a higher proportion of female suicidal ideations as compared to males, in proportion to the USN population demographics, *X*^2^(1, *N* = 6291) = 305.41, *p* < 0.001. For age, more servicemembers aged 25 and under displayed suicidal ideation compared to other groups, which constitutes 70.58% of the total reported incidents from 2011 to 2020, *X*^2^(4, *N* = 6247) = 2272.55, *p* < 0.001. Regarding paygrades, there were significantly higher reported suicidal ideations in the paygrades of E1–E3, *X*^2^(3, *N* = 6175) = 1741.88, *p* < 0.001. For years 2011–2018 with the Pacific Fleet data, there was no statistically significant difference between white and black sailors in proportion to the overall demographics. However, for years 2019 and 2020 with the merged Pacific Fleet and Atlantic Fleet data, there was a significantly higher proportion of suicidal ideations reported for black sailors.

**Table 3:** Suicide risk behaviors: distribution of selected characteristics for the total sample and by demographics across the years

	Pacific Fleet (2010–2018)																	
	2010		2011		2012		2013		2014		2015		2016		2017		2018	
	N	%	N	%	N	%	N	%	N	%	N	%	N	%	N	%	N	%
Suicide ideations																		
Gender			*p* *<* *0.001*		*p* *<* *0.001*		*p* *<* *0.001*		*p* *<* *0.05*		*p* *<* *0.001*		*p* *> 0.05*		*p* *<* *0.001*		*p* *<* *0.001*	
Female			115	23.61	117	22.81	106	24.54	110	22.22	108	30.00	93	22.09	130	27.96	123	30.00
Male			372	76.39	396	77.19	326	75.46	385	77.78	252	70.00	328	77.91	335	72.04	287	70.00
Other			2						2									
Age			*p* *<* *0.001*		*p* *<* *0.001*		*p* *<* *0.001*		*p* *<* *0.001*		*p* *<* *0.001*		*p* *<* *0.001*		*p* *<* *0.001*		*p* *<* *0.001*	
25 and under			340	70.54	343	67.25	301	70.33	373	75.20	265	73.61	304	72.38	309	66.45	296	72.20
26–30			83	17.22	90	17.65	81	18.93	77	15.52	55	15.28	63	15.00	95	20.43	73	17.80
31–35			35	7.26	43	8.43	23	5.37	20	4.03	26	7.22	29	6.90	30	6.45	23	5.61
36–40			20	4.15	24	4.71	19	4.44	18	3.63	11	3.06	18	4.29	20	4.30	13	3.17
41+			4	0.83	10	1.96	4	0.93	8	1.61	3	0.83	6	1.43	11	2.37	5	1.22
Other			7		3		4		1				1					
Race			*p* *> 0.05*		*p* *> 0.05*		*p* *> 0.05*		*p* *> 0.05*		*p* *> 0.05*		*p* *> 0.05*		*p* *> 0.05*		*p* *> 0.05*	
White			319	80.96	319	78.38	268	75.49	321	80.05	229	78.97	292	78.49	307	76.94	288	78.90
Black			75	19.04	88	21.62	87	24.51	80	19.95	61	21.03	80	21.51	92	23.06	77	21.10
Other			95		106		77		96		70		49		66		45	
Rank			*p* *<* *0.001*		*p* *<* *0.001*		*p* *<* *0.001*		*p* *<* *0.001*		*p* *<* *0.001*		*p* *<* *0.001*		*p* *<* *0.001*		*p* *<* *0.001*	
E1–E3			217	46.67	178	36.03	176	41.71	206	42.92	138	39.20	181	42.99	161	34.62	161	39.27
E4–E6			227	48.82	289	58.50	225	53.32	249	51.88	199	56.53	208	49.41	267	57.42	224	54.63
E7–E9			9	1.94	14	2.83	13	3.08	15	3.13	4	1.14	14	3.33	15	3.23	12	2.93
Officer/CWO			12	2.58	13	2.63	8	1.90	10	2.08	11	3.13	18	4.28	22	4.73	13	3.17
Other			24		19		10		17		8							
Suicide attempts																		
Gender			*p* *> 0.05*		*p* *<* *0.001*		*p* *> 0.05*		*p* *> 0.05*		*p* *<* *0.01*		*p* *<* *0.001*		*p* *<* *0.001*		*p* *<* *0.01*	
Female			13	19.12	38	34.23	4	17.39	7	23.33	17	32.08	20	38.46	42	39.62	28	32.56
Male			55	80.88	73	65.77	19	82.61	23	76.67	36	67.92	32	61.54	64	60.38	58	67.44
Other																		
Age			*p* *<* *0.001*		*p* *<* *0.001*		*p* *> 0.05*		*p* *> 0.05*		*p* *<* *0.001*		*p* *<* *0.001*		*p* *<* *0.001*		*p* *<* *0.001*	
25 and under			52	78.79	80	72.07	16	69.57	15	51.72	39	73.58	39	75.00	83	78.30	61	70.93
26–30			7	10.61	17	15.32	4	17.39	4	13.79	13	24.53	11	21.15	14	13.21	13	15.12
31–35			7	10.61	7	6.31	2	8.70	3	10.34	1	1.89	1	1.92	4	3.77	4	4.65
36–40			0	0.00	4	3.60	0	0.00	5	17.24	0	0.00	1	1.92	4	3.77	5	5.81
41+			0	0.00	3	2.70	1	4.35	2	6.90	0	0.00	0	0.00	1	0.94	3	3.49
Other			2						1									
Race			*p* *<* *0.05*		*p* *> 0.05*		*p* *> 0.05*		*p* *> 0.05*		*p* *> 0.05*		*p* *> 0.05*		*p* *> 0.05*		*p* *> 0.05*	
White			37	64.91	67	72.04	16	84.21	20	74.07	29	74.36	30	66.67	72	77.42	56	76.71
Black			20	35.09	26	27.96	3	15.79	7	25.93	10	25.64	15	33.33	21	22.58	17	23.29
Other			11		18		4		3		14		7		13		13	
Rank			*p* *<* *0.001*		*p* *<* *0.001*		*p* *> 0.05*		*p* *> 0.05*		*p* *<* *0.001*		*p* *<* *0.001*		*p* *<* *0.001*		*p* *<* *0.01*	
E1–E3			33	49.25	51	46.36	7	30.43	9	31.03	21	39.62	24	46.15	50	47.17	30	34.88
E4–E6			33	49.25	55	50.00	14	60.87	15	51.72	30	56.60	27	51.92	54	50.94	46	53.49
E7–E9			0	0.00	2	1.82	2	8.70	3	10.34	1	1.89	0	0.00	1	0.94	1	1.16
Officer/CWO			1	1.49	2	1.82	0	0.00	2	6.90	1	1.89	1	1.92	1	0.94	9	10.47
Other			1		1				1									
Suicides																		
Gender	*p* *> 0.05*		*p* *> 0.05*		*p* *> 0.05*		*p* *> 0.05*		*p* *> 0.05*		*p* *> 0.05*		*p* *> 0.05*		*p* *> 0.05*		*p* *> 0.05*	
Female	1	7.69	0	0.00	0	0.00	1	10.00	0	0.00	1	11.11	0	0.00	1	6.67	3	14.29
Male	12	92.31	10	100.00	12	100.00	9	90.00	14	100.00	8	88.89	16	100.00	14	93.33	18	85.71
Other			1															
Age	*p* *> 0.05*		*p* *> 0.05*		*p* *> 0.05*		*p* *> 0.05*		*p* *> 0.05*		*p* *<* *0.001*		*p* *> 0.05*		*p* *> 0.05*		*p* *> 0.05*	
25 and under	8	61.54	0	0.00	6	50.00	4	40.00	5	35.71	6	66.67	7	43.75	5	33.33	12	57.14
26–30	2	15.38	4	40.00	2	16.67	4	40.00	2	14.29	2	22.22	4	25.00	6	40.00	3	14.29
31–35	1	7.69	2	20.00	3	25.00	1	10.00	3	21.43	1	11.11	3	18.75	2	13.33	2	9.52
36–40	2	15.38	3	30.00		0.00	0	0.00	4	28.57		0.00	1	6.25	2	13.33	1	4.76
41+		0.00	1	10.00	1	8.33	1	10.00		0.00		0.00	1	6.25		0.00	3	14.29
Other			1	1.47														
Race	*p* *> 0.05*		*p* *> 0.05*		*p* *> 0.05*		*p* *> 0.05*		*p* *> 0.05*		*p* *> 0.05*		*p* *> 0.05*		*p* *<* *0.05*		*p* *> 0.05*	
White	9	81.82	7	70.00	7	77.78	8	100.00	12	100.00	4	57.14	13	86.67	14	100.00	15	93.75
Black	2	18.18	3	30.00	2	22.22	0	0.00	0	0.00	3	42.86	2	13.33	0	0.00	1	6.25
Other	2		1		3		2		2		2		1		1		5	
Rank	*p* *> 0.05*		*p* *> 0.05*		*p* *> 0.05*		*p* *> 0.05*		*p* *> 0.05*		*p* *> 0.05*		*p* *> 0.05*		*p* *> 0.05*		*p* *> 0.05*	
E1–E3	2	15.38	0	0.00	2	16.67	1	10.00	1	7.14	5	55.56	2	12.50	3	20.00	4	19.05
E4–E6	9	69.23	7	70.00	9	75.00	7	70.00	9	64.29	3	33.33	11	68.75	8	53.33	13	61.90
E7–E9	0	0.00	1	10.00	0	0.00	1	10.00	0	0.00	1	11.11	2	12.50	2	13.33	2	9.52
Officer/CWO	2	15.38	2	20.00	1	8.33	1	10.00	4	28.57	0	0.00	1	6.25	2	13.33	2	9.52
Other			1															

	Pacific and Atlantic Fleet (2019–2020)						
	2019		2020		Total incidents		Navy average demographics 2010–2020
	N	%	N	%	N	%	%
Suicide ideations							
Gender	*p* *<* *0.001*		*p* *<* *0.001*		*p* *<* *0.001*		
Female	264	30.95	520	28.03	1686	26.80	18.26
Male	589	69.05	1335	71.97	4605	73.20	81.74
Other	6		15		25		
Age	*p* *<* *0.001*		*p* *<* *0.001*		*p* *<* *0.001*		
25 and under	590	70.91	1288	69.85	4409	70.58	42.07
26–30	149	17.91	324	17.57	1090	17.45	23.06
31–35	51	6.13	154	8.35	434	6.95	15.21
36–40	25	3.00	56	3.04	224	3.59	10.86
41+	17	2.04	22	1.19	90	1.44	8.81
Other	27		26		69		
Race	*p* *<* *0.01*		*p* *<* *0.001*		*p* *<* *0.001*		
White	464	73.30	981	71.19	3788	75.85	78.06
Black	169	26.70	397	28.81	1206	24.15	21.94
Other	226		492		1322		
Rank	*p* *<* *0.001*		*p* *<* *0.001*		*p* *<* *0.001*		
E1–E3	323	38.68	663	36.21	2404	38.93	22.57
E4–E6	470	56.29	1063	58.06	3421	55.40	51.40
E7–E9	27	3.23	46	2.51	169	2.74	9.30
Officer/CWO	15	1.80	59	3.22	181	2.93	16.73
Other	24		39		141		
Suicide attempts							
Gender	*p* *<* *0.01*		*p* *<* *0.001*		*p* *<* *0.001*		
Female	39	31.71	74	38.54	282	33.41	18.26
Male	84	68.29	118	61.46	562	66.59	81.74
Other							
Age	*p* *<* *0.001*		*p* *<* *0.001*		*p* *<* *0.001*		
25 and under	92	75.41	137	73.66	614	73.62	42.07
26–30	18	14.75	29	15.59	130	15.59	23.06
31–35	4	3.28	16	8.60	49	5.88	15.21
36–40	5	4.10	2	1.08	26	3.12	10.86
41+	3	2.46	2	1.08	15	1.80	8.81
Other	1		6		10		
Race	*p* *> 0.05*		*p* *<* *0.01*		*p* *<* *0.001*		
White	70	73.68	98	67.59	495	72.16	78.06
Black	25	26.32	47	32.41	191	27.84	21.94
Other	28		47		158		
Rank	*p* *<* *0.001*		*p* *<* *0.001*		*p* *<* *0.001*		
E1–E3	45	37.19	77	40.53	347	41.46	22.57
E4–E6	69	57.02	105	55.26	448	53.52	51.40
E7–E9	4	3.31	4	2.11	18	2.15	9.30
Officer/CWO	3	2.48	4	2.11	24	2.87	16.73
Other	2		2		7		
Suicides							
Gender	*p* *> 0.05*		*p* *> 0.05*		*p* *<* *0.001*		
Female	2	9.52	4	10.81	13	7.30	18.26
Male	19	90.48	33	89.19	165	92.70	81.74
Other					1		
Age	*p* *> 0.05*		*p* *> 0.05*		*p* *> 0.05*		
25 and under	12	57.14	15	55.56	87	48.88	42.07
26–30	6	28.57	4	14.81	41	23.03	23.06
31–35	1	4.76	6	22.22	25	14.04	15.21
36–40	2	9.52	2	7.41	17	9.55	10.86
41+		0.00		0.00	8	4.49	8.81
Other					1		
Race	*p* *> 0.05*		*p* *> 0.05*		*p* *<* *0.01*		
White	15	93.75	26	86.67	130	87.84	78.06
Black	1	6.25	4	13.33	18	12.16	21.94
Other	5		7		31		
Rank	*p* *> 0.05*		*p* *> 0.05*		*p* *<* *0.05*		
E1–E3	8	38.10	9	24.32	37	20.79	22.57
E4–E6	10	47.62	24	64.86	110	61.80	51.40
E7–E9	1	4.76	3	8.11	13	7.30	9.30
Officer/CWO	2	9.52	1	2.70	18	10.11	16.73
Other					1		

#### Suicide attempts

There were a total of 282 female suicide attempts (33.41%) as compared to 562 attempts for males (66.59%) across all years, which was significantly higher in proportion to the demographics (18.26% and 81.74%), *X*^2^(1, *N* = 844) = 129.29, *p* < 0.001. With respect to age, there were many more attempted suicides for those aged 25 and under (73.62%), in proportion to the USN’s population demographics (42.07%), *X*^2^(4, *N* = 834) = 359.43, *p* < 0.001. For each individual year, in general, suicide attempts across white and black sailors were consistent to the population, but when aggregated, there were more suicide attempts among black sailors, *X*^2^(1, *N* = 686) = 13.97, *p* < 0.001. Regarding rank, once again, suicide attempts occurred more frequently amongst those in the E1–E3 paygrades in proportion to the USN’s demographics, *X*^2^(3, *N* = 837) = 275.43, *p* < 0.001.

#### Suicides

When assessing suicides by year in proportion to the USN’s overall demographics, no statistically significant differences were observed between females and males. However, when aggregated across 2010–2020, there were more male than female suicides, *X*^2^(1, *N* = 178) = 14.36, *p* < 0.001. Regarding age, there was no evidence of a robust difference in the proportions of reported incidents and the proportions expected from the overall USN population, *X*^2^(4, *N* = 178) = 6.22, *p* > 0.05. For by year assessments, no statistically significant differences between white and black sailors were observed, but aggregating the data longitudinally resulted in a reliably greater suicide number in white than in black sailors, *X*^2^(1, *N* = 148) = 8.25, *p* < 0.01. Regarding paygrade, there was no statistically significant difference across paygrades, but when aggregated from 2010 to 2020, there were slightly more suicides in E4–E6 and fewer among Officers, *X*^2^(3, *N* = 178) = 9.40, *p* < 0.05.

#### Suicide odds-ratio

Logistic regression models were constructed to determine the relationships between suicidal attempts and suicide ideations based on key demographics (i.e., gender, age, race, and paygrade). Gender was significant at the 0.05 level with an OR of 0.80 (95% CI 0.66–0.98). Males were 20% less likely than females to attempt suicide versus exhibit suicidal ideation. For age, the ORs by age category in reference to the age under 25 group: 26–30 (OR 0.82; 95% CI 0.64–1.05), 31–35 (OR 0.82; 95% CI 0.55–1.20), 36–40 (OR 1; 95% CI 0.58–1.72), and > 41 (OR 1.12; 95% CI 0.54–2.32), and there was no significant difference by age. Regarding race, white sailors exhibited an OR of 0.87 (95% CI 0.69–1.10) to attempt suicide, but it was not significantly different from black sailors. For paygrade, those E4–E6 had an OR of 0.78 (95% CI 0.64–0.94), E7–E9 had an OR of 0.61 (0.33–1.11), and Officers had an OR of 0.70 (95% CI 0.4–1.19). E4–E6 had a significantly lower OR (*p* < 0.05) as compared to the reference group E1–E3, in terms of attempting suicides versus suicidal ideations.

## Discussion

Consistent with previous research [13], males committed the majority of domestic violence incidents in the USN population understudy. However, the relative proportion in the sample did not differ from the expected relative proportions of males and females in the overall USN population, which supports findings from previous studies of mixed gender military populations [24]. This study’s findings suggest that generically assuming that males are de facto perpetrators should thus not be the modus operandi; instead, a more comprehensive taxonomy of acts of aggression in the military context should be developed to better inform prevention and intervention efforts.

Concerning sexual assaults, males were overwhelmingly reported as being offenders in the majority of incidents. It is noteworthy that the proportion of males and females is quite different in sexual assault incidents compared to domestic violence incidents. As such, these destructive behaviors should not necessarily be classed together as male aggressions. Each behavior likely requires unique research attention in order to better understand them.

Regarding the relational dynamics associated with sexual assaults, the finding that offenders were three times more likely to outrank the victim sheds light on a facet of the social constructs underlying such incidents. This is also in contrast to the relational dynamics in domestic partners, which occurred more in the same ranks. This could be because of the definition of domestic partners as being an intimate partner or adult family member, but it could also shed light into the nature of sexual assaults, such as these aggressions manifesting when there is a difference in power or social position [21]. Indeed, differences in power may explain why younger, junior servicemembers may be at greater risk for sexual assault (in addition to factors such as living on base in close quarters [30]). These results suggest that prevention efforts could be targeted towards specific ranks to offset potential perpetrators, while other prevention efforts could be designed for lower ranks to enhance potential victim awareness. Additional research is needed to tease apart the contributions of these different factors.

Alcohol was often cited in domestic violence reports and its use was frequently associated with the offender and the victim, which conforms to previous substance use research [29, 55]. In particular, underage drinking poses an important problem, especially as seen in the sexual assault and domestic violence cases.

In terms of suicide behaviors, in proportion to the USN population demographics, females were more likely to exhibit suicidal ideation and suicide attempts, which is consistent with previous findings [56]. Various theories account for such gender differences, whether it is because it is more acceptable for females to express a perceived vulnerability or because they are more likely to use a suicide attempt as a means of communicating distress [57]. In terms of actual suicides, males represented the majority of completed suicides, which is also in line with previous research that has found that men are more adept with fatal weapon use [58].

In terms of age, suicidal ideations and attempts were higher for sailors aged 25 and younger, which aligns with previous results of people at a younger age being more at-risk [59]. However, there was no statistically reliable difference in the actual suicides carried out by the age groups in proportion to the Navy demographics. This suggests that military suicide intervention efforts should continue to target young servicemembers.

Regarding race, for the 2011–2018 Pacific Surface Fleet data, suicidal ideations and attempts for white and black sailors were roughly in proportion to USN population demographics (78.06% and 21.94%). For the larger 2019–2020 dataset (Pacific and Atlantic Surface Fleets), there was a significantly greater number of ideations and attempts amongst black sailors. However, for completed suicides, there was a significantly greater number of suicides by white sailors aggregated across the years. The greater number of suicides by white sailors is aligned with previous results, both in military and general population studies, but the high number of ideations and attempts in black sailors in recent years warrants further research to explore the interplay between race and suicidal ideation, suicide attempts, and actual suicides.

Regarding rank and suicide, sailors in the paygrades E1–E3 exhibited more suicidal ideations and attempts relative to other ranks. However, there were significantly more completed suicides in the E4–E6 paygrades than would be expected in the general USN population demographics. As there is little extant research that explores relationship between rank and suicide behaviors, more research in this area is warranted.

In conclusion, this study presents the findings from unique longitudinal destructive behavior dataset from 2010 to 2020. The study provides an overview of the possible factors associated with these behaviors and explores the relational dynamics and nature of the incidents. The results help inform the development of prevention and mitigation efforts. A noteworthy finding is the relationship between paygrade differences and sexual assaulters and victims; although it warrants additional research, this finding suggests a two-pronged intervention strategy whereby prevention efforts should target higher paygrades to offset potential perpetrators and interventions designed to enhance potential victim awareness might be directed at more junior paygrades.

In all, this study leverages a decade’s worth of unique data to document the prevalence rates of maladaptive behaviors in at-risk naval force populations and provides a contextual understanding of the underlying factors. To supplement these quantitative insights, analysis should be conducted on the qualitative nature of such incidents to better illuminate more specific candidate causes of destructive behaviors within military populations. In particular, interviews can uncover the challenges faced by servicemembers across the various ages, gender, race, and ranks, and further inform the development of policies, practices, and targeted interventions.

### Strengths and limitations

This study’s strength lies in leveraging a unique longitudinal destructive behavior dataset. The study also compares the incident reporting data to the USN’s overall demographics data for the past decade to identify trends and also help interpret the findings.

The data are focused specifically on the USN’s Surface Force; as such, it does not reflect destructive behavior incidents across the entire USN. Another limitation is that given the nature of the incident reports, the data are subject to incomplete data (e.g., a victim’s information might not be available at the time the report is submitted) and inaccurate or inconsistent data categorization given that multiple people file the reports. Also, OPREP-3 derived data only reflect events that matriculate to a command’s attention; thus, these findings might not fully reflect all events.

There were also many unknown and missing values, due to the complex and sensitive nature of the incidents, thus the actual number of cases could perhaps be underreported. However, these are challenges and limitations faced by most applied research. Despite the limitations, this study provides an overview of incident report data spanning a decade which illuminates domestic violence, sexual assault, and suicide risk destructive behaviors in a unique military population.

## Data Availability

Access to the data may be provided by submitting a Freedom of Information Act request via https://www.cnic.navy.mil/foia/foia_request.html.

## Abbreviations

DoD: Department of Defense
OR: Odds ratio
OPREP-3: Operational Reports
US: United States
USN: United States Navy
